# Addressing COVID-19 Testing Inequities Among Underserved Populations in Massachusetts: A Rapid Qualitative Exploration of Health Center Staff, Partner, and Resident Perceptions

**DOI:** 10.3389/fpubh.2022.838544

**Published:** 2022-03-24

**Authors:** Rebekka M. Lee, Veronica L. Handunge, Samantha L. Augenbraun, Huy Nguyen, Cristina Huebner Torres, Alyssa Ruiz, Karen M. Emmons, for the RADx-MA Research Partnership

**Affiliations:** ^1^Department of Social and Behavioral Sciences, Harvard T.H. Chan School of Public Health, Boston, MA, United States; ^2^DotHouse Health, Dorchester, MA, United States; ^3^Caring Health Center, Springfield, MA, United States; ^4^Lynn Community Health Center, Lynn, MA, United States

**Keywords:** COVID-19 testing, community health centers, structural inequities, qualitative, needs assessment, rapid analysis, immigrant populations

## Abstract

**Introduction:**

Access to COVID-19 testing has been inequitable and misaligned with community need. However, community health centers have played a critical role in addressing the COVID-19 testing needs of historically disadvantaged communities. The aim of this paper is to explore the perceptions of COVID-19 testing barriers in six Massachusetts communities that are predominantly low income and describe how these findings were used to build tailored clinical-community strategies to addressing testing inequities.

**Methods:**

Between November 2020 and February 2021, we conducted 84 semi-structured qualitative interviews with 107 community health center staff, community partners, and residents. Resident interviews were conducted in English, Spanish, Vietnamese, and Arabic. We used a 2-phase framework analysis to analyze the data, including deductive coding to facilitate rapid analysis for action and an in-depth thematic analysis applying the Social Ecological Model.

**Results:**

Through the rapid needs assessment, we developed cross-site suggestions to improve testing implementation and communications, as well as community-specific recommendations (e.g., locations for mobile testing sites and local communication channels). Upstream barriers identified in the thematic analysis included accessibility of state-run testing sites, weak social safety nets, and lack of testing supplies and staffing that contributed to long wait times. These factors hindered residents' abilities to get tested, which was further exacerbated by individual fears surrounding the testing process and limited knowledge on testing availability.

**Discussion:**

Our rapid, qualitative approach created the foundation for implementing strategies that reached underserved populations at the peak of the COVID-19 pandemic in winter 2021. We explored perceptions of testing barriers and created actionable summaries within 1–2 months of data collection. Partnering community health centers in Massachusetts were able to use these data to respond to the local needs of each community. This study underscores the substantial impact of upstream, structural disparities on the individual experience of COVID-19 and demonstrates the utility of shifting from a typical years' long research translation process to a rapid approach of using data for action.

## Introduction

Since the detection of the first COVID-19 case in the U.S. in January 2020, the country has surpassed over 50 million COVID-19 infections and more than 800,000 deaths (1). Higher social vulnerability has been associated with higher COVID-19 case fatality rates (2). National and state level data indicate that the COVID-19 pandemic has disproportionately affected Black and Latinx populations in the U.S., with these groups experiencing higher rates of infection, hospitalization, and mortality (3, 4)^1^. Black populations have had up to 3.5 times the risk for infection compared to White populations, and Black and Latinx populations have had three times the risk of mortality from COVID-19 compared to white populations (5). Inequities in COVID-19 infections and deaths have also been identified in Native American, Asian American, and Pacific Islander populations. Additionally, U.S. counties with more immigrants have also been found to have more COVID-19 cases (6).

These patterns highlight how structural racism, defined as “the totality of ways in which societies foster racial discrimination through mutually reinforcing systems of housing, education, employment, earnings, benefits, credit, media, health care, and criminal justice” (7), perpetuates health inequities in many communities of color (7–9). In particular, these disparities are reflective of the social determinants of health and comorbidity rates that further affect the level of burden of COVID-19 in these populations (10). Communities with poorer housing conditions (e.g., overcrowded, high housing cost burden) are at higher risk for COVID-19, in part due to their inability to isolate (11). Additionally, racial/ethnic minorities are over-represented in essential jobs that increase exposure to the virus (12, 13). Furthermore, poor treatment by healthcare institutions and the government, both in the past and currently, has negatively impacted trust with communities of color and subsequently affected adherence to COVID-19 public health recommendations (14). Unsurprisingly, the highest COVID-19 death rates in Massachusetts have been observed among those living in the most disadvantaged areas based on zip code tabulation area data (i.e., poverty level, household crowding, percentage population of color, and racialized economic segregation) (15). For these populations, recommended prevention and mitigation strategies such as social distancing are often not feasible.

The inequitable patterns of COVID-19 disease burden have highlighted the urgent need for improved testing, surveillance and monitoring, data transparency, and targeting of public health interventions to the circumstances in which people live, work, play, and pray. In particular, access to testing in underserved communities is imperative for combatting the COVID-19 virus. Yet, throughout the pandemic, testing access has often been inequitable and not aligned with community need. Communities of color and low-income communities have had less access to testing, and as a result have had lower testing rates. Studies in both New York City and Massachusetts indicate that COVID-19 testing has been much more accessible in neighborhoods that are more affluent and with larger White populations, even though COVID-19 exposure as well as the proportion of positive tests has often been higher in more racially diverse neighborhoods and areas with lower socioeconomic status (16, 17).

Prior studies have explored residents' perceived barriers to both coping with COVID-19 and COVID-19 testing. Barriers to testing identified include lack of access to testing, confusion about testing guidelines and eligibility, social stigma and concerns about the consequences of testing positive, cost of testing, logistics including transportation to testing sites, and fear and mistrust (18, 19). In order to address these barriers, public health and healthcare institutions have partnered with community-based organizations (e.g., churches, schools) and community health centers to increase COVID-19 testing and COVID-19 communications in under-resourced communities (20–23).

Community health centers (CHCs), “community-based and patient-directed organizations that deliver comprehensive, culturally competent, high-quality primary care services…regardless of patient's ability to pay” (24)^2^, have been on the front lines of the pandemic. CHCs disproportionately serve low-income populations (91% of patients) and people of color (over 60% of patients) (25). Early in the pandemic, CHCs were facing significant financial loss due to the drop-off in primary care visits, while at the same time they took on the challenge of scaling up public testing. For a variety of reasons, most private primary care providers did not engage in testing, and testing was largely provided by CHCs and governmental agencies. Nationally, ninety percent of CHCs have been providing COVID-19 testing and over half (57%) of people tested at these sites are people of color (25)^3^. The Commonwealth of Massachusetts also rolled out a free “Stop the Spread” testing program in July 2020 with mass testing sites across the state (26). At the peak of the pandemic, the statewide initiative included over 35 sites (26); however, as of the end of 2021, the program continues at 10 sites across the state (27)^4^. There is limited research linking barriers to COVID-19 testing with solutions, particularly the role of CHC partnerships to address these challenges. Additionally, there is limited research on the unique barriers to testing faced by people with limited English proficiency and immigrant populations in the U.S.

Given the widespread need for COVID-19 testing support, following Congress-approved funding in April 2020, the National Institutes of Health (NIH) created the Rapid Acceleration of Diagnostics (RADx) program. The aim of the RADx program is to develop innovative diagnostic technologies and strategies to increase testing access. One of the four RADx programs is Rapid Acceleration of COVID-19 Testing in Underrepresented Populations (RADx-UP), which aims to understand factors that have led to the disproportionate burden of the pandemic on underserved populations and to support improved access and uptake of COVID-19 testing through community-engaged efforts (28).

The RADx-UP program funded our project in Massachusetts (RADx-MA), one of over 70 RADx-UP projects throughout the U.S. This project is led by a collaboration between the Harvard T.H. Chan School of Public Health, Massachusetts General Hospital, and the Massachusetts League of Community Health Centers and is supported through the infrastructure of the Implementation Science Center for Cancer Control Equity (ISCCCE). It builds on existing and new partnerships between CHCs and community organizations in six Massachusetts COVID-19 hotspot communities with both high rates of illness and racial/ethnic gaps. The aim of this project is to work with the CHC-community partnerships to develop expanded testing implementation strategies and to conduct a series of community-engaged pilot studies to assess the impact of different approaches to addressing barriers to testing. To prepare for the planned work supporting implementation of testing strategies to reach underserved populations, we conducted a rapid, comprehensive needs assessment with stakeholders at multiple levels. In this paper, we explore the perceptions of COVID-19 testing barriers among community health center staff, community partners, and residents gathered through this rapid needs assessment. We also describe how these community-identified needs and assets can be translated to build tailored clinical-community strategies for addressing testing inequities.

## Materials and Methods

### Qualitative Interview Design, Recruitment, and Consent

This needs assessment utilized 30-min qualitative semi-structured interviews to gather a broad range of perspectives across six Massachusetts communities. First, in November 2020, the lead author conducted interviews with staff members at nine participating community health centers. Next, from November 2020 to February 2021, the lead author and two research assistants used a snowball sampling approach to conduct interviews with staff and volunteers from organizations the health center staff identified as current or future partners in COVID-19 testing. Types of organizations interviewed included community coalitions, local boards of health, housing authorities and shelters, food banks, and immigrant advocacy groups. Finally, from December 2020 to February 2021, four additional research assistants conducted resident interviews in English, Spanish, Vietnamese, and Arabic, reflecting primary languages in the participating communities. Separate interview guides (see Appendix A) for staff, partners, and residents were developed with parallel questions to capture actionable feedback to inform changes to testing practices and communications. Study staff worked with health center and partner organization staff to recruit a convenience sample of community residents for interviews, distributing recruitment flyers in four languages through newsletters, social media, and via online community meetings. Residents contacted study staff by phone or email to learn about the study activities, risks and benefits, and to schedule the interview. Verbal consent detailing the study purpose, logistics, and confidentiality was obtained prior to the start of each interview and a written consent script was also distributed to participants via email or text. The study was approved by the Harvard Longwood Campus Institutional Review Board.

### Qualitative Interview Procedures

All interviews were conducted using semi-structured interview guides with parallel questions for each audience about the experience of COVID-19 testing in their community, perceived barriers to testing, communication and education needs, perspectives on mobile and rapid testing strategies, and local successes. Community health center staff and partners were also asked about strategies for partnership to improve access to, utilization of, and communication about testing. Individuals were compensated $25 for participation in interviews. Interviews were conducted over Zoom, recorded, and transcribed verbatim. Prior to analysis, transcripts of interviews conducted in Spanish, Vietnamese, and Arabic were translated into English by the bilingual research assistants who conducted the interviews.

### Qualitative Analysis

**Figure 1:**
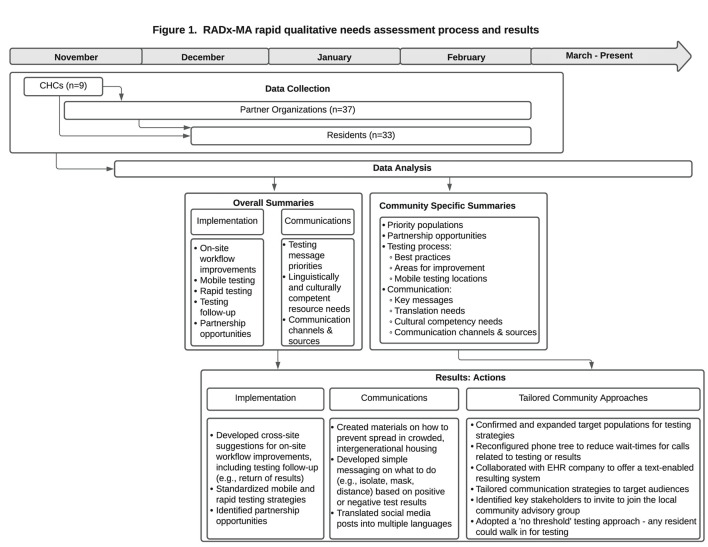
RADx-MA rapid qualitative needs assessment process and results.

We conducted a 2-phase framework analysis (29) to facilitate rapid return of results (30, 31) and utilization of data for action (Figure 1). First, two coders (SA and VH) categorized and summarized content from health center and partner interviews to share with the RADx project testing implementation team and communication team in January 2021. In March 2021, community-specific summaries from health center, partner, and resident interviews were developed for local action.

In the second phase, the research team conducted an in-depth thematic analysis, deductively coding interviews into the five levels of the Social-Ecological Model (policy and environmental; community; organizational; interpersonal; individual) (32) according to the framing described by the interviewees and into three pre-figured codes drawn from the interview guide (testing process, communications, and partnerships). This was followed by inductive coding to develop constructs and sub-constructs within these five levels. This framework was selected to emphasize the multilevel influences on COVID-19 testing experience. A codebook was developed and shared with other members of the research team to gather feedback and define agreed upon constructs. The same coders proceeded to double-code 16 interviews, reconciling codes, and revising the codebook in consultation with the senior author as appropriate. The remaining transcripts were single coded, divided between the two coders. Analyses were conducted using NVivo qualitative data analysis software Version 11 and then summarized and condensed into salient themes.

## Results

**Figure 2:**
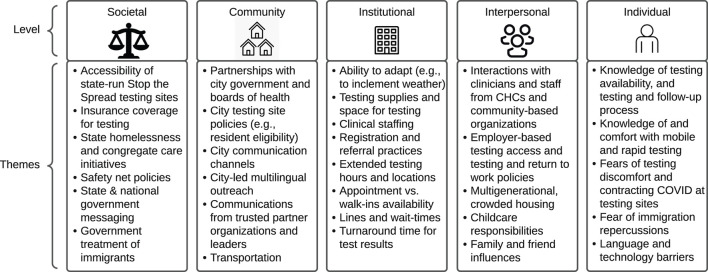
COVID-19 testing barriers & facilitators themes organized by the social ecological framework.

Findings from the qualitative analysis are organized by phase. The Phase 1 action-oriented qualitative process and results are presented in Figure 1. The Phase 2 thematic analysis is organized by theme within the five levels of the Social Ecological Model (32), starting with the societal level to emphasize the structural determinants described by participants (see Figure 2; Tables 1–5). Similar themes were often identified among health center staff, partners, and residents. However, when discordant views emerged between groups, a detailed description of these differing perspectives is provided. Exemplar quotes within each theme are also displayed in tables by role. A total of 107 individuals - community health center staff (*n* = 12), partners (*n* = 57) and residents (*n* = 38) - across 84 interviews, participated in the interviews and are included in both phases of the results.

**Table 1:** Societal factors influencing COVID-19 testing reported by community health center staff, partners, and residents.

**Theme**	**Quotes**
State-run Stop the Spread sites are open to everyone regardless of insurance coverage, referral, or symptoms, and are free of charge	“*Obviously the Stop the Spread campaign adds a layer of financial security because it's absolutely free for people, whereas at the health center, we will bill you if you have insurance.” (CHC)* “*I work at City Hall where the Stop the Spread site - one Stop the Spread site is, and they're open every, almost every day unless there's inclement weather and they're always busy… I think everybody knows that they can come and get free testing.” (Partner)* “*I went to a Stop the Spread* [site]*, because once you get tested once with a* [hospital] *clinic, they don't like to reschedule appointments…. My son and myself tested negative at first. And then I called them back, saying that everyone in my household had tested positive and that I just wanted to get retested the 4-5 days after just to make sure that we were still negative to continue quarantining appropriately, or if we were positive that way we could stop quarantining…I had to go to a Stop the Spread site in* [another city]”* (Resident)*
State-run Stop the Spread sites are not enough to address testing inequities	“*We are still interested in looking to reach [undocumented immigrants and refugees], since they may miss a lot of the traditional messaging, like about Stop the Spread - that's in English.” (CHC)* “*I mean, there were certain things, like Stop the Spread came into existence that that wasn't our doing. That was the state trying to appease, you know… trying to make accessible testing in a way that made sense to them. But from our perspective, it's not enough and it's not flexible enough and it's not targeted enough to actually make an impact for the vulnerable populations that we're interested in.” (Partner)*
Insurance coverage impacts testing	“*One thing that's come up is when we're referring just like asymptomatic patients that just want to get tested because they want to go see grandma… not necessarily a positive exposure, we're running up a little bit against whether insurance will cover their test” (CHC)* “*I think for the community… [testing through partner organizations] will identify families who may have COVID that may not be going to hospitals, may not be seeking health care… they don't have insurance, so by the time they seek health care, they're really sick” (Partner)* “*I thought I was going to go into one of those clinics, and I just did not know how much I was going to be charged or if I was going to be charged. I still don't know because it's kind of hard to get on the phone with [my insurance] about the cost” (Resident)*
State initiatives support access to testing for underserved populations	“*Because many of our programs are residential and congregate care programs, so we were required to test 50% of our staff in a two- week period because of the positivity rate. Our agency next week, officially by state mandate, will go up to testing 100%. But we also [only] have the ability to get reimbursed for one test per resident per month.” (Partner)* “*Well, yes, homeless people. The state is starting something that is partially responding to that. They're making available rapid tests. But from what I understand, the rapid tests are available for people who are symptomatic. That's who that test responds to. And I think we worry about how much asymptomatic spread there is.” (Partner)*
Need for better safety net policies for people who test positive	“*How much money do we have? Can we pay everybody for seven days off of work when they get their diagnosis? I mean, that's a federal policy kind of thing. But, you know, that's, I think a big driver of this, right? It's fear of a positive diagnosis. Having to lose work for two weeks is just a huge I think that's a big issue.” (CHC)* “I think people have exhausted some of their benefits. So if you test positive, the government will give you the 80 hours of paid time in home.* We see people on the second wave. So they might have had some sort of exposure or positive test [in] *April, May, and June. So they used up that bank of 80 hours and now testing might be more available than it was in May and June. But they don't want to know even more so now, because that same hesitation they had in the summer that they didn't want the inconvenience or the monetary consequence of not being able to work overtime. Now it's ‘I'm not going to get paid at all.' ”* (Partner) *Note: this refers to those who qualify through federal leave under FFCRA, the CARES Act, and ARPA
Messaging from state and national government impact testing	“*If you look around the country…all these people are just being crazy, but we're not providing them with clear messaging on what to do.” (CHC)* “*It's really difficult for us to mobilize our community to go to do the testing because they saw the news saying that no need to do tests, no need to do masks, so that's a lot of misinformation and the country was very divided at that time. It's a very confusing chaotic thing because we don't have good leadership…in the [Centers for Disease Control]…they are the one who's supposed to make the final decision…” (Partner)* “*I think that if the government were able to disseminate the information through social media - because it feels like the whole world is connected through social media…then more people would have access to [testing] and feel more confident about doing it.” (Resident)*
Need for more collaboration across health systems and between state and local entities	“*It has been helpful that the state has put together excellent materials that people like us and [CHC] and others can then take and amplify in our own ways. So I think the partnership between state and local entities could not be more important because they need to use us in better ways to spread the word” (Partner)* “*What would be amazing, that's just been lacking everywhere is some kind of a streamlined overall approach… having a situation where the MA League [of Community Health Centers] or your organization figures out ok, what does this need to look like to do this everywhere? How do you register it into the system? How does it get coordinated with all the different EHRs? The problem is in our health care system, it is unbelievably fragmented” (Partner*)
Government treatment of immigrants impacts testing	“*There's so much fear around immigration and this miserable administration.” (Partner)* “*Circling back to the immigration piece… People have a legitimate fear of being deported. And so there's a lot of questions that people have of whether getting a free test is somehow a benefit that will be considered a public charge.” (Partner)*

**Table 2:** Community factors influencing COVID-19 testing reported by community health center staff, partners, and residents.

**Theme**	**Quotes**
Boards of health are integral for COVID-19 response	“*I have reached out a couple of times to the… board of health nurse… In the beginning, some patients weren't allowed to get tested because of they didn't meet certain criteria. And then I would refer those patients over to her and they had testing for everyone.” (CHC)* “*Certainly the shelters know to immediately contact our Department of Health… It helps too that our largest shelter, has a health clinic within the building. So there is a very close connection to health care and to the Department of Health, which is one of the funders of that health clinic” (Partner)*
Partnerships with city government strengthen COVID-19 response	“*If any health centers and cities or towns in which they're located can have the kind of support and relationship that we have with [our] city… right now, it's been it's been foundational for our ability to mount the response that we've mounted.” (CHC)* “*One of the things that we started, I helped start, back in March* [2020] *was… the* [City-run] *COVID Community Response Network and where it's a coalition of different community leaders, community organizations, faith leaders, and we created different subgroups, part of the network, so we have a wellness team that we made phone calls to over 8000 of our seniors and some of our residents in housing properties. And so we're creating a neighborhood pod system” (Partner)* “[The city] *is very much saturated with nonprofits that already have connections to the community and ways to get in touch with community members. And so I say use the infrastructure that's already there within City Hall and those other nonprofits and ensuring that those that are most affected in the process, black and brown individuals, are the ones that are leading those efforts as well.” (Resident)*
City policies related to residency impact testing eligibility	“*We had two Stop the Spread sites. And then when one of them shut down, the city opened up that, like, super site in* [the race track]. *And that's for everybody, not just for [our city] residents.” (CHC)* “*And now we have a [city] resident only testing site, but not everybody is accessing or knows about them” (partner)* “*Truthfully, I just don't feel like* [the testing site] *was servicing just the people of* [our city] …* they were coming from* [small suburban city]*, they were coming from [town], They were coming from* [another city] *to come here to our town to get a test because we set up some sites and they didn't have their own. They maybe had one testing site or you had to go, like I said, to the urgent care. So not only were we not servicing our own people fully, but then we start servicing everybody else …And that's how you're contributing to the four hour line” (Resident)*
Cities communicate COVID-19 information through various channels	“*The mayor's office has, like their communications team, has been helpful in our wording of these like emails. I got to see the newsletters, things like that. So we have their communications team, which has been helpful in getting advertising out for like town halls” (CHC)* “*Part of also the ambassador program, we work with* [City public access] *TV and we have a weekly PSA type of TV show. So we produce videos and all of the major challenges. And so that information is getting out. We do get a lot of views on those videos.” (Partner)* “*You know, I think that the communication with* [the city] *has been nice. We are signed up for their alert system, so and I follow their Facebook. So like I was aware that the testing was out there.” (Resident)*
City-supported multilingual initiatives impact testing	“*This morning I was watching the mayor's COVID update and although it's great, it's only in English. So it's really hard for our patient population or our patients to kind of follow everything that's going on” (CHC)* “*We've been leading the communication efforts for the city and then with the ambassador program, which we launched towards the end of August* [2020]*, which is a team of multilingual speaking residents who are doing community engagement work with those who have been impacted by COVID” (Partner)* “*I also know that in* [the city] *there have been really great efforts, both official through City Hall and volunteers, and they have been mobile sites and there have been efforts to get out multilingual material.” (Resident)*
Limited public facing communications on testing availability	“*Having messaging around about its availability would actually help because I think one of the barriers is people just don't know where to get tested. Myself as a provider, like, I feel like it's changing every week - where I'm supposed to be sending my patients?” (CHC)* “*You know, I think with the biggest barriers still, you know, not everybody knows that* [the city] *has all these different testing sites available… We had two, one at the beach and then one at the high school, which is still running. And we're looking to run until the end of March* [2021]. *But people still don't know about these testing sites.”(Partner)* “*There wasn't really a central location where she could find where all the testing locations were at and where to preregister. So I worked at City Hall previously, and so I am familiar with the* [city] *website and contacted a couple of people to get additional information. But I did notice if someone wasn't in the know or had access to someone within City Hall, it's really difficult to find a central location where all testing sites can be found in addition to any preregistration link.” (Resident)*
Trusted community leaders promote COVID-19 communications	“*I think there is an inherent trust…I think that could be there, that might be accessed by having these ambassadors who are not just working for the city, but are kind of peers and live in the city and kind of have that trust” (CHC)* “*A key aspect around getting more people tested in the community, it's really kind of finding those leaders in the community, those residents who have built rapport and relationships that people trust and rely on to be able to be leaders and set the example is important.” (Partner)* “*So I've had other friends who needed* [testing] *and they've been like, ‘oh my God, there's nowhere in the state where I can get tested.' And it's like, oh, hey, here's the information. I've had that for them. So I think that I feel well connected to* [the city's] *system… And I let my neighbor know about them.” (Resident)*
Transportation impacts access to testing	“*Well, I guess if you don't have a vehicle, you don't have transportation, that's going to be challenging because if you don't live near the testing sites and a lot of our patients do rely on public transportation and recently the* [city transit system] *had a press statement that they had to reduce services because of their own staff.” (CHC)* “*If you're talking about a vulnerable population who doesn't have access to transportation or their own car, and you're faced with the same situation where they're either not getting tested or they're taking public transportation to get a test. Yeah, so that's that kind of seems to be the biggest barrier.” (Partner)* “*They offered free testing at that location. Well, I couldn't go unless I pulled up in a car. And I thought to myself. ‘This is not good for people in my neighborhood…' A lot of people don't have cars, so they walk and they take the bus and they take the train to go to the supermarket or whatever their daily needs are.” (Resident)*

**Table 3:** Institutional and Organizational factors influencing COVID-19 testing reported by community health center staff, partners, and residents.

**Theme**	**Quotes**
Organization's capacity to adapt (e.g., to inclement weather) positively impacts testing	“*We have pivoted our wellness center, which is a seventeen hundred square foot space in one site to become an isolation unit and to be our covid testing indoor space. So people who are getting tested go around those doors and come in that way.” (CHC)* “*And a lot of that funding came…from the city of Boston and the Boston Public Health Commission that just allowed us to purchase an all-weather cubicle that we considered during the winter and heaters and try to figure out like a tarp covering things so that we can have our drive-thru site to continue during the winter. So we offer every day testing at our site.” (Partner)*
Limited access to supplies hinders ability to offer rapid testing	“*Yes, we signed an agreement to bring rapid molecular testing in-house. It's just not available. So the manufacturer, Abbott, told us that they're backorder. And, you know, at least through the end of the year, we will not be getting machines or reagent.” (CHC)* “*And during the last week, since now it's been since March. So April, May, June, July, August, September. So for eight months, we only had access to supplies for the PCR test… We were not able to obtain any rapid testing supply. And that to us was a huge disparity and inequity in the distribution of resources across Massachusetts. And we have been advocating strongly with anyone who will listen about how community health centers need to get rapid testing” (Partner)*
Limited staffing is a barrier to testing	“*You see the results pile up and you have to like pull staff from places. It's really hard to just find the staffing… the tent is struggling with having enough registration staff… And it's a whole other service line that came out of nowhere that isn't going anywhere. And it's just kind of like struggling staffing wise to kind of keep up with resulting and everything that goes along in testing and all the positions that go along with making that happen” (CHC)* “*You know, [CHC] has, like, a lot of culturally competent staff, but they're also very overwhelmed.” (Partner)* “*So I don't know if they're understaffed, but I've had to wait [on hold] up to 45 minutes… Sometimes they hang up on you or you have to hang up.” (Resident)*
Varied registration and referral practices impact testing experience	“*We have our patients calling our front desk and they're waiting. Then they're getting a message sent to the nurse, then the nurses connecting with one of us providers that we're weighing in on whether the patient needs to get testing, then the testing, then the nurse is calling back the patient. Then the testing is like - it's like so many steps.” (CHC)* “*So notifications were super important then: we gave [the hospital] all of our residents for them to preregister, so that help*[ed] *on the administrative side of things.” (Partner)* “*And that was a barrier, too, because you're supposed to call your primary care doctor, and then they're supposed to give you a referral to get a testing. And then I was very upset because to get a referral from your doctor, you have to get a telemedicine visit. And I'm like, then that costs you money to get a telehealth visit, right?” (Resident)*
Consistent, extended testing hours and locations improve testing uptake	“*So I think definitely accessibility, like the hours as you had mentioned before. A lot of our patients or a lot of our residents are considered essential workers, but they're still going into work, commuting into work. So having a more expanded testing hours, I think it would be helpful… more evening hours.” (CHC)* “*This is one of the other big, big things: you have to have consistent everyday hours, even on the weekend, because we found… it's on Tuesday and this place Thursday, it's in this place on Wednesdays. And like a lot of [people say] ‘I just need to know exactly where to go.' ” (Partner)* “*And another challenge for a lot of people, even some people in my family, was that they worked during the week so they couldn't go. They couldn't go to the free walk*[in] *ones, they couldn't go to those on the weekends because they were not available on the weekends. It was usually like either like a Monday or a weekday. So that was a challenge.” (Resident)*
Preferences for appointments vs. walk-in testing varied	“*The community members out there found us off the city… website and noticed that we were the only ones that, one of the few, … that don't require an appointment yet. That's why you chose to come here, which is great.” (CHC)* “*I think the walk ins would be a lot, much more easier for clients. Like if I could say, ‘hey, you want to go wait on this line for a minute, you know, get testing knocked out and, you know, you get your results and it'll be safer for you and everybody that, you know that is around you.”' (Partner)* “*What I've noticed that there are a lot of people out there who get desperate when it comes time to make the appointment on the phone and go for the walk-in. But if they're already full, they can't walk-in.” (Resident)*
Long lines and wait-times are a barrier to testing	“*The long lines* [are] *creating a barrier… Our messaging was so good that everyone's testing, but now we've shot ourselves in the foot because we have tremendously long lines” (CHC)* “*Right around Thanksgiving, I would say the lines were really out of control for these sites. And so I don't know how much awareness of that there was. But you made the decision to getting tested. You ended up being in a line of cars” (Partner)* “*We tried going through, like, Stop the Spread sites and everywhere was closed or the places that were open have lines of like a thousand people where like some people were in line for like four or five hours already and they had barely moved” (Resident)*
There is a delay in turnaround time for testing results	“*Turnaround time is a…very significant barrier to the utility of a test…*[if] *it takes more than more than 48 hours to get back means it's in many ways it's a waste. It's a waste test, because …., people don't tend to actually socially… isolate themselves from family members and the people that they're at risk from spreading to until they actually have the result.” (CHC)* “*But at this point, I think the biggest barrier is the wait time for results. Like rapid testing was something that there was a lot of talk around that we were going to get access to. But there haven't really been any places close by in order to get [it].” (Partner)* “*There was someone who did travel and we needed to get it. They need 72 hours or so to travel. Checking that the result was done… If it's a little late, Three, four days… that really doesn't give you much room to say get the result and be able to get out of town.” (Resident)*
Demand for testing outpaces supplies and resources	“*We get much more requests for testing that we can even accommodate.” (CHC)* “*Since the testing blew up, [the CHC] was getting so many test requests from the community that they almost couldn't fit our people in. (Partner)*

**Table 4:** Interpersonal factors influencing COVID-19 testing reported by community health center staff, partners, and residents.

**Theme**	**Quotes**
Interactions with clinical staff impact testing uptake and experience	“*Yeah, I mean, I think as providers, we could probably also do a better job of just being able to have more people around the table… just talk to our families and talk to people we serve around their thoughts about being tested. Have they been tested? And why or why not?.” (CHC)* “*A lot of times they're told by their doctor like, ‘oh, you probably have it, but you don't need to get tested'. There's been a lot of that, like telehealth, where our young people have been told, OK, so they have symptoms, but, you know, they should just quarantine and they don't need to get tested.” (Partner)* “*Thank God they spoke well to me. And I was a little scared, wasn't I? Because they says they are tough with you, but thank God not the person who did it to me they had a good hand because I didn't, I didn't feel it.” (Resident)*
Clinicians are a trusted source of COVID-19 related information	“*We had a few Spanish speaking doctors who would come in and talk and answer some questions for community members … just trying to get a better sense of what was what was rumor and what was real.” (Partner)* “*My doctor would definitely be able [to help me]. I mean, he's like, I think the head doctor at the health center and he's been there using my doctor for good almost 30 years. So, you know, I trust him. And he's like very knowledgeable.” (Resident)*
Well known, trusted staff at CHCs and CBOs promote testing	“*We have, interestingly, had a great deal of success with specific staff. Our numbers for like our requests for testing have gone up… the week after [the trusted staff member] retweeted something, or if they share something on Facebook.” (CHC)* “*Our staff have good credibility with folks and they go out trying to help. So they go out with food and we've given them blankets…And we kind of lead with all that help. And along the way, you know, we're also talking to them about whatever. And we might suggest to them that they get tested. (Partner)*
Employers enable access to testing	“*We had a staff person who was outreach to every business. They were reopening and just saying, ‘if you need testing, let us know'…we don't do that actively [now], because now we're known.” (CHC)* “*And I know a lot of our families are in this work in the service industry, like we have a lot of folks who work down at Whole Foods, who work at Dunkin Donuts and who work for Amazon and many of those companies. And I think many of those places may be required to have more of a rapid testing situation.” (Partner)* “*Yes. My husband tests regularly, he works at Amazon and they offer testing on site where he works” (Resident)*
Employer policies for return to work not aligned with public health guidance	“*And I think one of the barriers is, like, work-related because of so many of…their employers are unforgiving and…they're doing things that don't even make sense, like they're asking people to get retested after they're positive and there's no protocol for that..” (CHC)* “*We've heard a lot. A lot, a lot, a lot about people having to get another test that says they're going to be able to go back to work, which is not true or it's not scientifically what needs to happen. But that's what a lot of employers in some places are requiring.” (Partner)* “*But since we saw that I had tested positive, then he took time off work and commented at work that I was sick. Then they told him that he couldn't go to work for at least three weeks, that he had to take the test and then go back to work. He had to take a negative.” (Resident)*
Testing can threaten job stability	“*We're aiming to also address folks who are at a substantial employment risk where the concept of having a test is the difference between retaining their job or not.” (CHC)* “*They're worried about today* [thinking], ‘*so if I go get tested today and it comes out positive today and I can't go to work'. And even though we would say your employer needs to offer you these benefits protection…there was I think a lack of trust that that would actually work” (Partner)* “*He missed* [work] *the day he went and then as they said he had to wait until he got the results… Four days he missed and since he only work five days a week, sometimes four. Then he lost the whole week.” (Resident)*
Work conflicts with testing access	”*They have a break from their job as an essential worker. So like a half an hour and they got go drive back. So we have to fix that.” (CHC)* “*Well I think time is always a barrier, and I say that because our families are probably working full-time jobs in the service industry there, sometimes two to three jobs to make ends meet. Our older kids are also working. And so taking the time to go get tested is something that's not on everybody's radar.” (Partner)* “*Like it's super inconvenient and people have to work, which is why the other people in my family weren't able to get as tested, tested as frequently as I would, because they'd get out of work, try to go. And it's like, oh, ‘we can't take any more people, we don't have enough tests' ” (Resident)*
Childcare responsibilities are a barrier to testing access	“*This is a barrier, like if you make it so that they only have a babysitter for an hour and they can go back and they can't wait in this line” (CHC)* “*As a parent, I'm thinking if I have three kids and I'm a single mom getting three kids in the car and going to the health center to get screening, especially on appointment, is going to be really difficult and challenging, especially if they're kids under the age of five.” (Partner)* “*The hard part is because the kids are in school and online class from home and when you have the kids during the school day it's too hard… he needs one person to be in the house. You cannot leave him by himself.” (Resident)*
Inability to isolate in crowded and multigenerational housing	“*I think they're seeing a lot of multigenerational households and people who are just feeling like, well, ‘I need to go to work and I need to put food on the table, and send my child to day care'. And ‘I'm going to go to work even if I have cold symptoms'. So they're just noting that the cases are becoming a little more complex because people are choosing with symptoms to go do those things because they feel like they have to, you know, to pay rent and all of that.” (CHC)* “*A lot of families live really close to other families and they have a two-bedroom house and have four or five people. And it's really hard to quarantine in a house that small where the ventilation is known to be really, really poor for families.” (Partner)* “*At one point we were one in four, one in four of our residents is COVID positive and continually cumulatively, it's the highest hit area and there's a reason for it right. And it's not because everyone here is just sickly, I mean, it's just because we're the hard working community and people want to work and it's overcrowding and there's a reason for it.” (Resident)*
Family and friends influence testing uptake	“*People within this community talk in a lot of it is through word of mouth and relationships. And I think when they hear somebody talk about their experiences, it may deter them from actually knowing about it and doing something on their own.” (Partner)* “*My mom is an essential worker. She works as a janitor as well. And so she's had to go in person to work since March. And a couple of our coworkers had tested positive. And so I had to encourage my mother to go get tested when that did happen. And luckily, she's been negative every time” (Resident)*
Stigma associated with testing positive is a barrier to testing	“*Right. I think that's the one of the bigger ones, literally just about to face stigma around positives.” (CHC)* “*And then in the early stages, I don't know if it's still the same, but there was like there was a feeling that we heard from community members that if they tested positive, people were sort of looking down at them like, ‘oh, you got COVID because you're dirty' or ‘you got COVID because of X', like there was a stigma put on folks who tested positive for COVID.” (Partner)*

**Table 5:** Individual factors influencing COVID-19 testing reported by community health center staff, partners, and residents.

**Theme**	**Quotes**
Limited knowledge of testing availability	“*I think one of the barriers is people just don't know where to get tested. Myself as a provider, I feel like it's changing every week - where I'm supposed to be sending my patients. I can't even keep track of it.” (CHC)* “*But even like we saw when [the CHC] came here this summer, you know, there weren't as many people as there should have been, could have been. I and I think it was all because of the word of mouth that hadn't gotten out.” (Partner)* “*Not everyone will know where the locations are at in their cities…I hear people all the time asking, posting things, asking, you know, where can we find a location that's available? How do we know the times that they're available?” (Resident)*
Limited knowledge of the testing and follow-up process	“*Other issues were with patients understanding of what was happening at that site and… when they could expect results.” (CHC)* “*When should you get tested? Like if, you know, you've been exposed or not tested or only if you're having symptoms? And how? When - right away* [or] *when you're having symptoms or when? So I think I find that a little confusing.” (Partner)* “*I think that was the first hurdle for my sister was to one, find out where can I find a close covid testing location. And then afterwards, do I need a preregister or can I just walk in?” (Resident)*
Limited knowledge of and comfort with mobile testing	“*We all noticed that if you are not consistently* [at] *place for like more than two weeks, it doesn't even yield a lot of patients.” (CHC)* “*And one of the things that comes up with mobile testing is locating testing in places that feel more accessible, that are more trusted.” (Partner)* “*At first, there was a doubt I also had was that how accurate could it be considering I wasn't in a health care facility, a hospital, or clinic. I was just like, OK, I didn't know how accurate it would be because I was like I said, it was it was totally a different set up for me. And that's something I've never been through was testing or something like that outside.” (Resident)*
Limited knowledge of and comfort with rapid testing	“*How accurate is the rapid testing? Because I hear you can get it back at 15, 20 minutes.” (Partner)* “*I know someone. She got the results ah, almost the next day. But she still said that she felt that it was not safe, the test. Because as it normally takes 3 to 4 days, receiving the next day is suspicious.” (Resident)*
Language barriers in accessing testing	“*I keep thinking they are Spanish primary speakers - like they just don't know the first even step of how to get assistance” (CHC)* “*Some people who have the language barrier, that makes things a lot a lot harder for people not to just get to the testing sites, understand it, to know what's happening, to even understand the results.” (Partner)* “*It would be helpful to have translators onsite to help with testing because people who don't speak English turn away from places that don't have their native speakers.” (Resident)*
Technological barriers in accessing testing information	“*Technology is a big barrier as well for some people. You and I might be tech savvy…when you go to sign up for one of these covid testing sites, you and I would register online… If you're not tech savvy and don't know how to go around the computer, how does that communication happen? How do you get that done?” (Partner)* “*I was having trouble getting into the internet…We cannot blame any worker or the system, but we have to stay updated. It's a bit more complicated for us because we were not used to the technology.” (Resident)*
Discomfort or fear associated with the testing process	“*We actually started to use the anterior nasal swab and so that…barrier has been reduced… But, you know, I honestly don't think it feels that much different, but a lot of people do. And I think psychologically it makes a huge difference.” (CHC)* “*It can be it can be very scary that when we get this test done. You know, as I say, that they put that thing right up into your brain for crying out loud. But it is. But it can be scary.” (Partner)* “*When people were talking about how horrible it felt to have this thing stuck up in their head scratching their brain… I had sinus surgery, actually, like literally the week before the lockdown…so hearing about people's experiences predisposed me to feel like I don't want to get tested unless I have to” (Resident)*
Fear of contracting COVID-19 at testing sites	“*I've had a few in the last few weeks that I've been on the phone. [I've told them] ‘you just you need to come in and get tested' or ‘you need to come into the clinic for other reasons that are unrelated to COVID testing'. And they're like, ‘I don't care. I'm not going anywhere near that building because COVID people go there.' ” (CHC)* “*My people I know like they wouldn't go to the hospital or center because they're afraid they might be exposed to COVID-19.” (Partner)* “*When I was standing in line, I feel really unsafe because I felt like I could be picking up COVID-19 from being in line because not everybody's conscientious about keeping the six foot distance. So, you know, I was worrying about being there in line myself.” (Resident)*
Mistrust in health system	“*People go where they feel comfortable, where they feel safe. So, especially in behavioral health, I think that a lot of people are very paranoid. They're very mistrustful, they've had trauma. They've had such bad experiences either in the health care system or otherwise” (Partner)* “*There was some fear of being quarantined and [the hospital] turning them into guinea pig for testing” (Resident)*
Fear of immigration-related repercussions	“*The governor created contact tracing; some of our residents may not have been as honest to them, whether it be because they just didn't want their work to find out, you know, maybe they were afraid being fired, maybe they had some fear of ICE or something.” (CHC Staff)* “*People have a legitimate fear of being deported. And so there's a lot of questions that people have of whether getting a free test is somehow of a benefit that will be considered a public charge.” (Partner)*

### Phase 1—Data for Action

Figure 1 provides an overview of the data collection process and results from Phase 1. Codes related to implementation of testing by the CHCs were used to develop cross-site suggestions for on-site workflow improvements, mobile testing strategies, rapid testing strategies, testing follow-up (e.g., return of results) strategies, and partnership opportunities. Summaries of the suggested strategies were provided to study staff to work directly with the CHCs to rapidly implement changes to the testing process. Codes related to COVID-19 communications captured priorities for messaging (e.g., topics of confusion), linguistically and culturally competent resource needs, and suggestions for communication channels (e.g., fliers, social media) and sources (e.g., faith-based leaders). These communications summaries were provided to study staff specializing in communications to develop simple messages in multiple languages for CHCs to disseminate to patients and partners. Community-specific analyses captured updated priority populations and partnership opportunities, testing areas for improvement, specific mobile testing site recommendations, key messages for tailored communications, translation needs, and suggested local communication channels and sources. Examples of how data were used for local action include:

- Interview data was used to **confirm and expand target populations for testing strategies**. Segments of the population prioritized included culturally and linguistically diverse populations, individuals with disabilities, individuals experiencing housing insecurity and homelessness, as well as those living in multigenerational housing where isolating may not be feasible when exposed to the virus.
- Interview data from patients helped **reveal an operational challenge in a CHC contact center**. Residents reported long wait times when calling one CHC to inquire about testing or results. Using this information, the CHC reconfigured their phone tree to put COVID-19 testing and resulting as the first two prompts, which shortened the wait time for patients. Separating COVID-19 from non-COVID-19 calls early in the call process allowed the CHC to connect patients with the correct staff and reduce the time it takes to receive COVID-19 results or to make an appointment for COVID-19 testing.
- Patients' descriptions of **challenges receiving timely results prompted the CHCs to address these challenges** internally and externally. Another CHC worked with their electronic medical record systems to offer a text enabled resulting system. Another CHC successfully advocated directly with the test vendor to prioritize specimens given the high prevalence rate in its community.
- Interview data helped the CHCs **tailor communication strategies to their target populations**. Partner organizations suggested that one CHC employ different channels of communication to connect with members of the community. In response, the CHC hosted COVID-19 discussion sessions at local businesses, popular gathering spots, and places of worship. They also employed methods such as paper and virtual fliers, video postings on social media, as well as regular updates on the CHC website.
- Partner interview data was used to **identify key external stakeholders to be invited to join the local community advisory group**. One CHC included leadership from a local shelter in its community advisory group. The CHC also partnered with this shelter to host mobile COVID-19 testing and vaccination events to reach individuals experiencing homelessness.
- Interview data was used to better understand **the need for mobile and static testing strategies and possible locations or frequency**. These topics became the agenda content of the local community advisory group for one CHC early in the project.
- Interviews revealed that **individual CHCs' testing eligibility criteria was confusing and difficult to navigate** for patients. This moved a CHC to adopt a “no threshold” approach: any resident could walk in for testing, without an appointment, whether they were a CHC patient or not and whether they were symptomatic or not.
- Interviews revealed that **the fear of being billed for a COVID-19 test**, even for insured individuals, was an obstacle. One health center looked for other avenues to get paid for testing when a patient was uninsured or when their insurance denied payment. This included using grant funding or billing the state's uninsured portal.

### Phase 2—Thematic Analysis

The major themes identified in this qualitative analysis are displayed in Figure 2. Tables 1–5 and narrative summaries below further explore these themes in greater depth with exemplar quotes from each stakeholder group (e.g., CHC staff, partners, residents) corresponding to the theme in each table.

#### Societal

CHC staff, partners, and residents across multiple communities described the state-run Stop the Spread mass vaccination sites as influential for accessing testing (33)^5^. These sites were open to everyone regardless of insurance coverage, referral, or symptom during the peak months of the pandemic and were free of charge. However, health center staff and partners expressed that these sites were not enough to address testing inequities and were particularly limited in terms of language accessibility. In addition to these public testing sites, partners described how separate state initiatives for congregate care settings and homeless shelters supported access to testing for underserved populations and the staff working in these settings. Another societal factor mentioned by all interviewee types was the impact of insurance coverage on testing—both lack of insurance and uncertainty in reimbursement were frequently mentioned as deterrents to seeking care. CHC staff and partners also discussed the need for better safety net policies for people who test positive to be able to take paid time off from work to stop the spread of the virus. Finally, many participants described how unclear messaging from state and national government hindered testing uptake, and partners emphasized that government mistreatment of immigrants (e.g., fears of Immigrant and Customs Enforcement) further exacerbated these challenges.

#### Community

CHC staff, partners, and residents described partnerships with city government as important to strengthening the COVID-19 response. City communications channels such as email newsletters, public access TV, and social media pages were described as important for disseminating COVID-19 education in multiple languages. However, public facing information about testing availability—locations and hours—was reportedly difficult to find. Stakeholders in cities where state mass vaccination sites were located also described how city policies related to residency impacted testing eligibility. Because state Stop the Spread sites were flooded with people from nearby cities and towns, those who lived in the city often struggled with access. Creating rules to limit city-run sites to local residents helped improve testing access. Transportation was another community level barrier to testing mentioned by CHC staff, partners, and residents—those without access to a vehicle could not access drive-thru testing sites and riding public transit to distant sites could increase risk of exposure. Finally, trusted community leaders—both individuals and community-based organizations—were described as important for promoting COVID-19 communication.

#### Organizational

Institutional and organizational factors influencing COVID-19 testing primarily focused on infrastructure of the CHCs. CHC staff and partners described the ability of the facilities to adapt their testing space as a facilitator for testing, particularly for inclement weather as the pandemic surged in the winter months. Barriers frequently mentioned were limited staffing, long lines and wait times, and delayed turnaround times for testing results. At the peak of the pandemic, the demand for testing also outpaced supplies such as machines and reagent to perform rapid testing. Interview participants reported varied experiences with registration and referral practices that impacted the likelihood of seeking testing—expressing a clear preference for an approach that is simple. Patients viewed the referral process that was sometimes required outside of CHC settings as adding extra time and cost burdens to seeking testing. Participants also expressed mixed experiences and preferences for COVID-19 testing with appointments vs. walk-in—with the suggestion to provide both options. CHC staff, partners and residents all agreed that consistent, extended testing hours and locations improved testing uptake.

#### Interpersonal

CHC staff, partners, and residents described interpersonal interactions with clinicians and community-based organization staff as positive influences on testing uptake and as trusted sources of information. Employers both enabled access to testing with on-site accessibility and hindered testing with return-to-work policies that did not align with public health guidance and threatened job stability. Family and friends were reported as influencing testing uptake and crowded, multigenerational housing often hindered the ability for residents to isolate in an attempt to mitigate spread.

#### Individual

Interviews uncovered limited knowledge of the testing and follow-up process, testing availability, mobile testing, and rapid testing. This translated to some discomfort with the ideas of mobile and rapid testing. Fears of the testing process and contracting COVID-19 at testing sites were also expressed. Individuals expressed how mistrust in the healthcare system and fears of immigration-related repercussions decreased the likelihood of residents seeking testing. Language barriers and challenges with technology needed to make appointments and receive timely results also limited access to testing.

## Discussion

This rapid, qualitative needs assessment of COVID-19 testing laid the groundwork for implementation of strategies to reach underserved populations at the peak of the pandemic in winter 2021. Using a 2-phase analysis process, we explored the perceptions of COVID-19 testing barriers among over 100 CHC staff, community partners, and residents. We created actionable summaries for the research team and CHC leaders to apply changes to testing strategies and address communication challenges immediately, prior to the completion of the theme identification process and full analysis. Additionally, this approach allowed for later more in-depth exploration of the multilevel factors influencing COVID-19 testing.

Massachusetts CHCs, along with the Massachusetts League of Community Health Center as a key advocate for needed policies and resources, were uniquely well-positioned to achieve the goal of the project to address COVID-19 testing inequities among underserved populations in “hotspot” communities. Their geographically identified service areas and majority patient-led and patient-engaged structure gave these CHCs the ability to use data to respond to the “hyper-local” needs of each community. This stands in contrast to the state mass vaccination sites that were very efficient, but unable to respond to the specific needs of underserved populations, particularly communities of color.

It is important to recognize that not all healthcare providers consider themselves to be a part of the essential public health infrastructure. As a result, when it comes to assuming risk to address a public health emergency like the COVID-19 pandemic, there are a number of critical questions that need to be addressed, such as who is accountable to the needs of the public's health—particularly the members of the public who are underserved? COVID-19 testing was about more than who had the resources to build and sustain this new service for residents. Setting up a testing program meant that staff members were at risk due to being exposed to potentially infectious individuals, and that staffing needs were increased, at a time when there was significant pressure on workforce availability. Developing a COVID-19 testing response was also incredibly disruptive to usual clinical care operations. As a result, the task fell primarily to healthcare institutions whose mission is to serve individuals and communities directly impacted by structural inequities, regardless of the cost or available resources. As we strive to learn how to do this better for the next pandemic or the next wave of this pandemic, do we make responsiveness to the community a criteria for public funding or non-profit status? Invest in government-run and tax-funded public health infrastructure? Or strengthen existing mission-driven community health partners like CHCs to be able to expand services/surge existing services to meet a public health need? Critical questions that must be answered are how do we build systems that are responsive to the needs of communities, and how do we mitigate structural barriers to care.

This qualitative study moves beyond individual perceptions of COVID-19 testing and underscores the substantial impact of upstream, structural disparities on the individual level experience of COVID-19, from infection to morbidity to mortality as well as on access to information and resources and uptake of preventive behaviors such as mask wearing and social distancing (19, 34). While we found that stakeholders expressed barriers and facilitators to testing at each level of the Social Ecological Model—individual, interpersonal, organization, community, and societal—the solutions for addressing these influences depend on institutional and governmental changes (32). Furthermore, while we discuss interpersonal factors such as the inability to isolate in crowded housing as circumstances of collective cultural household living, it is necessary to underscore that societal-level inequities (i.e., housing policies) create and perpetuate such factors in marginalized communities (35). The structural barriers identified, such as inadequate safety net policies for workers, immigration policies, and transportation, have lasting and multiplicative impacts at the individual level and may serve as launch points for next steps and future translational research. While prior studies have explored barriers and facilitators to COVID-19 testing among the general population or among one specific sub-population (18, 19, 36, 37), our study drew on a diverse array of perspectives to understand barriers and facilitators specific to several underserved populations (e.g., individuals experiencing homelessness, immigrant populations, individuals living in multigenerational households). This data better informed how CHCs and community partners could apply targeted approaches most relevant to specific populations disproportionally impacted by COVID-19.

Strengths of this study include its rapid approach to qualitative data collection and analysis that enabled the project to translate data for action in real time—this was essential for responding to a rapidly evolving pandemic. We were also able to interview 107 individuals across nine communities and in four languages—an approach that allowed for generating cross-cutting recommendations and identifying tailored community-specific needs. Building RADx-MA into our existing implementation science infrastructure in CHCs ensured that the project was uniquely health center-centric and responsive. Actionable response to findings was not prescribed to the CHCs, but rather the findings were provided to the health centers and each used the data to develop responses unique to their setting and to best meet the needs of their communities and resource structure. We shared results with CHC leaders and CABs to receive feedback on accuracy of data interpretation. Our CHC co-authors on this paper bolstered these perspectives, but we were unable to carry out member checking with residents. A trade-off with the breadth and action-oriented nature of the rapid needs assessment was our ability to capture demographics beyond the role and primary language of the participants. We were also only able to double-code a small sample of our interviews and did not have the staffing with the language skills to conduct interviews in Haitian Creole and Cape Verdean Creole—two language needs expressed by our CHC partners.

The lessons learned from this study were put to the test as the Omicron variant hit communities in December 2021. For instance, during the 2-week peak of the surge one CHC was able to accommodate an acute 10-fold increase in testing demand by pivoting back to the strategies identified through the rapid needs assessment and put in place earlier in the year. The CHC was able to quickly shift clinician resources to provide culturally and linguistically accessible positive result notification and medical evaluation of newly identified community residents with COVID-19 infection. Looking forward, these qualitative findings point to the importance of looking upstream to structural solutions at the organizational, community, and societal levels to prepare CHCs and the historically disadvantaged populations they serve for potential future surges and the endemic stage of the virus.

The COVID-19 pandemic has solidified the notion that health and social wellbeing are inextricably intertwined. In response to the pandemic, CHCs reached into the community to develop cross-sector partnerships that support health and wellness. Medical and non-medical drivers of health, and the relationship between them, should continue to gain recognition in the future of health care and offer insights into the social value of CHC's efforts to shape the health of communities. The present study lifts up examples of multilevel influences on health and demonstrates a shift from the typical years' long research translation process to a rapid approach of using data for action.

## Data Availability Statement

The raw data supporting the conclusions of this article will be made available by the authors, without undue reservation.

## Ethics Statement

The studies involving human participants were reviewed and approved by Harvard Longwood Campus Institutional Review Board. Written informed consent for participation was not required for this study in accordance with the national legislation and the institutional requirements.

## Author Contributions

KE and RL contributed to the conception and design of the study. RL participated in the data collection. VH and SA conducted the qualitative analysis. All authors participated in the data interpretation, drafted the manuscript for intellectual content, contributed to manuscript revisions, and approved the submitted version.

## Funding

This work was conducted with support from the National Institutes of Health (3P50CA244433-02S1), Harvard Catalyst | The Harvard Clinical and Translational Science Center (National Center for Advancing Translational Sciences, National Institutes of Health Award UL1 TR002541), and financial contributions from Harvard University and its affiliated Academic Healthcare Centers.

## Author Disclaimer

The content is solely the responsibility of the authors and does not necessarily represent the official views of Harvard Catalyst, Harvard University and its affiliated Academic Healthcare Centers, or the National Institutes of Health.

## Conflict of Interest

The authors declare that the research was conducted in the absence of any commercial or financial relationships that could be construed as a potential conflict of interest.

## Publisher's Note

All claims expressed in this article are solely those of the authors and do not necessarily represent those of their affiliated organizations, or those of the publisher, the editors and the reviewers. Any product that may be evaluated in this article, or claim that may be made by its manufacturer, is not guaranteed or endorsed by the publisher.

## Footnotes

^1^ https://www.cdc.gov/coronavirus/2019-ncov/community/health-equity/racial-ethnic-disparities/disparities-deaths.html

^2^ https://bphc.hrsa.gov/about/what-is-a-health-center/index.html

^3^ https://www.kff.org/coronavirus-covid-19/issue-brief/impact-of-coronavirus-on-community-health-centers/

^4^ https://transformativehc.com/stop-the-spread/

^5^ https://www.mass.gov/info-details/find-a-covid-19-test
