# Supplementary material for: Addressing COVID-19 Testing Inequities Among Underserved Populations in Massachusetts: A Rapid Qualitative Exploration of Health Center Staff, Partner, and Resident Perceptions
Source: Front Public Health. 2022 Mar 24;10:838544. doi: 10.3389/fpubh.2022.838544 (PMC8987278; doi:10.3389/fpubh.2022.838544)
Supplement: Supplementary file 1 [file Data_Sheet_1.PDF]

RADX Needs Assessment Questions – Community Health Center

Thank you so much for taking the time to speak with me. Today's conversation is meant to build on the needs assessment that we conducting during the proposal writing stage of the project and the meetings that were held with the team in October.

1. A major focus of this grant is to address testing inequities among vulnerable and underserved populations. You have previously shared that you would like to focus on addressing the needs of [insert population]. Are these still your target? Are there any other populations experiencing testing inequities in your community you would like to focus on?
    - Can you share how you identified these populations? (e.g. work with local Board of Health, monitor waste water, track demographics of patient population being tested)

2. You have previously shared that folks are experiencing the following barriers to testing [insert barriers]. Do these barriers still remain? What additional barriers have you noticed since the summer?

    - Probe for each population mentioned
    - What strategies have you tried to address to these barriers that seem to be working?
    - Any strategies that you would like to amplify?
    - What needs exist for engaging community members with limited English language? Written informational resources? Communication via non-English media about testing? Interpreters?
    - Any test reimbursement challenges? Payment for tests for asymptomatic people?

3. To address these barriers and inequities, we will be using a community-partnered infrastructure. You shared many useful details on your partnerships during the proposal stage, such as [insert examples]. Now we want to identify the specific partners that might be able to help support this project. What partner organizations should you engage with this month to reach these underserved populations?

    - In what ways do you see this partner helping to support outreach and testing?
    - Who at this organization would be best for you to speak with?
    - What ways do you think this grant will help strengthen your relationship?

4. Mobile testing (i.e. any testing that happens off site of a health center) has become one strategy health centers have moved towards to reach more underserved residents. During the October call, you told us that that [insert mobile testing knowns]. Is there anything else I should know about your experience with mobile testing?
    - Probe for successes and barriers
    - Probe to understand staffing

5. Rapid or point of care testing (i.e. same day antigen testing) has become another strategy health centers have moved towards to reach more underserved residents. During the October call, you told us that [insert rapid testing knowns]. Is there anything else I should know about your experience with rapid testing? [SKIP QUESTION IF CHC INDICATED THEY WERE NOT DOING POC BECAUSE THEY HAD GOOD PCR TURNAROUND]
    - Probe for successes and barriers
    - Probe to understand staffing

6. Partnerships with schools have also emerged as activities for reaching residents as classrooms have opened back up. During the October call, you told us that [insert school knowns]. Is there anything else I should know about your experience with school partnerships?
    - Probe for process, timeline, specific schools
    - Probe for successes and barriers
    - Do you have any similar partnerships with workplaces? Congregate living facilities? What successes or barriers have you seen there?

7. During the October call, you told us that [insert school knowns]. Is there anything else I should know about the plans you have for adapting your testing strategy for the winter months?
    - Probe for process & timeline
    - Probe to understand staffing

8. We've talked a lot about barriers to testing today, but we can often learn from successes. To conclude, do you have any local successes around addressing testing inequities you would like to share? Ideas that we might want to build upon?

    - What would you consider success in your community?
    - What synergies between CHCs could be leveraged to for shared learning? What would be helpful to know about other CHCs?
    - How could we have you achieve success?
    - Probe for Stop the Spread successes

RADX Needs Assessment Questions – Partner

Thank you so much for taking the time to speak with me. As you know, [insert CHC name] identified you as a potential partner for supporting the expansion of COVID-19 testing in your community. Today's conversation is meant to build what I've learned from them.

1. To get us started, can you share your role and the organization where you work/volunteer?

2. A major focus of this grant is to address testing inequities among underserved populations. The [insert community health center] has identified [insert populations] as the populations experiencing inequities in your community. What has been your experience working with these groups within your community?

3. Could you share the barriers to testing you think they face?
   - Probe for each population mentioned
   - Have you worked with them to try to improve access to testing?
   - What strategies have you tried to address these barriers that seem to be working?
   - Conversely, are that strategies that you have tried that have not worked out?
   - Any strategies that you would like to amplify?
   - What needs exist for engaging community members with limited English language skills? Written informational resources? Communication via non-English media about testing? Interpreters?
   - Any strategies that would also address the social needs of this population?

4. To address these barriers and inequities, we will be using community partnerships to support and strengthen outreach and engagement for testing. How do you see your role in a partnership aimed at increasing testing? How could this work help strengthen your relationship with the [insert name] community health center? What are some best practices you have for partnerships like this?

5. One aspect of this outreach is improving communications around testing. What kind of support do you think this grant could provide to better communication to [underserved population]? What messages or education to you think are most important to focus on?

6. We are exploring various innovations to try to increase access to testing in the community that I would like to get your thoughts on.

   - What is your experience with **mobile testing** in your community? By mobile testing, we mean any testing that happen outside of a hospital or health center or doctors office, such as at a community organization, worksite, or a van that tests in different parts of the city. How do you think it addresses the barriers to testing among underserved residents? Potential challenges?

- What is your experience with **rapid testing**? By rapid testing, we mean COVID testing that is done on the same day, often within just a few hours. How do you think it addresses the barriers to testing among underserved residents?

7. Are there any other populations experiencing testing inequities in your community you think we should know about as we plan this initiative?

8. We've talked a lot about barriers to testing today, but we can often learn from successes. To conclude, do you have any local successes around addressing testing inequities you would like to share? Ideas that we might want to build upon?

RADX Needs Assessment Questions – Resident

9. To get started, I'd love to learn a little about who you are. Can you share where you live and how you are connected to the [insert partner organization]?

10. What has life been like for you since COVID-19 hit last March?

11. Have you or anyone in your family been tested for COVID-19?

    - If yes, what was that experience like? Where did you go? Did you experience any challenges? Did you receive your results quickly?
    - If no, if you needed to be tested, what would you do?
    - If no, are there things that got in the way of being tested when you wanted to?

12. A major focus of this project is to make sure everyone in the community can be tested for COVID-19 if they need to be. Are you interested in being tested now? Why or why not?

13. Can you share any challenges to testing you, your family, or friends have experienced? What would make it easier for you to go get tested for COVID-19 if you needed to?

    - Do you have the information you need to figure out how to get testing? In your language? That is easy to understand? What additional information would be helpful?
    - Potential probes: time off work, lost wages, cost of testing

14. In what ways do you typically hear about health-related guidance, such as COVID-19 testing?

15. We are exploring different ways to make it easier to get tested.

    - What is your experience with **mobile testing** in your community? By mobile testing, we mean any testing that happen outside of a hospital or health center or doctor's office, such as at a community organization, worksite, or a van that tests in different parts of the city. Do you know anyone who has gotten a test at a van or other place like this? What do you think about it?
    - What is your experience with **rapid testing**? By rapid testing, we mean COVID testing that is done on the same day, often within just a few hours. Do you know anyone who has gotten a rapid test when they get results with just a few hours? What do you think about it?

16. To conclude, our hope is to help people like you in [insert community] be able to be tested for COVID-19 whenever they need it. Do you have any final suggestion for us to meet this goal?
